# An Efficient Hybrid Linear Clustering Superpixel Decomposition Framework for Traffic Scene Semantic Segmentation

**DOI:** 10.3390/s23021002

**Published:** 2023-01-15

**Authors:** Dan Zhong, Tiehu Li, Yuxuan Dong

**Affiliations:** 1 School of Automation, Northwestern Polytechnical University, Xi’an 710072, China; 2 School of Materials Science and Engineering, Northwestern Polytechnical University, Xi’an 710072, China; 3 School of Computer Science and Technology, Xidian University, Xi’an 710071, China

**Keywords:** superpixel decomposition, two-stage framework, linear clustering, online averaging

## Abstract

Superpixel decomposition could reconstruct an image through meaningful fragments to extract regional features, thus boosting the performance of advanced computer vision tasks. To further optimize the computational efficiency as well as segmentation quality, a novel framework is proposed to generate superpixels from the perspective of hybridizing two existing linear clustering frameworks. Instead of conventional grid sampling seeds for region clustering, a fast convergence strategy is first introduced to center the final superpixel clusters, which is based on an accelerated convergence strategy. Superpixels are then generated from a center-fixed online average clustering, which adopts region growing to label all pixels in an efficient one-pass manner. The experiments verify that the integration of this two-step implementation could generate a synergistic effect and that it becomes more well-rounded than each single method. Compared with other state-of-the-art superpixel algorithms, the proposed framework achieves a comparable overall performance in terms of segmentation accuracy, spatial compactness and running efficiency; moreover, an application on image segmentation verifies its facilitation for traffic scene analysis.

## 1. Introduction

Superpixel decomposition is essentially an unsupervised over-segmentation process that divides an image into several fragments without intersecting in the field of computer vision. It could reconstruct an image with fewer image patches, termed superpixels [1], which generalize pixel-wise primitives as region-level descriptors for subsequent processes. Many advanced visual tasks adopt it as a pre-processing tool by exploiting the potential to diminish the redundancy of input information, ranging from remote sensing [2], traffic analysis [3], geographic monitoring [4] and target detection [5].

Since it was first popularized in 2003, a growing number of classical superpixel algorithms have been put forward to improve the representation ability of visual information. To further distinguish from conventional image over-segmentation algorithms, several properties are imposed on superpixels, such as accuracy, efficiency and visual quality [6], wherein accuracy is the key property for superpixel generation, directly impacting the follow-up performance of visual tasks. This field mainly reveals boundary adherence. The efficiency directly judges whether a superpixel algorithm can be introduced as a pre-processing tool in several real-time applications. The distribution reports the appearance quality of produced superpixels, such as spatial topology and shape uniformity.

Nevertheless, there is still a contradiction between segmentation precision and visual quality in most superpixel generation methods. For example, there is seldom uniform visual information in real-world scenarios. On the contrary, such images usually consist of highly variable objects and textured backgrounds. In this case, the spatial compactness of superpixels is fragile, resulting in an unpleasant visual appearance [7].

To tackle this problem, simple linear iterative clustering (SLIC) [8] brings up a series of instructive optimizations to provide a balanced trade-off between these two properties. First, it adopts grid sampling to initialize clustering centers (also known as seeds), which could control the amount of superpixels as well as the clustering scope approximately [9]. Meanwhile, in the clustering period, it utilizes a restricted region inspection strategy to improve the efficiency of the heuristic method k-means approach [10]. Furthermore, and most importantly, the proposed joint color–spatial correlation measurement could both control the color homogeneity and shape compactness. As a result, these optimizations can yield a synergistic effect to generate satisfactory superpixels. 

More recently, a non-iterative version of SLIC is proposed in [11], which is also termed simple non-iterative clustering (SNIC). Compared with the conventional SLIC, it adopts an online averaging framework to remove the limitations that the label updating processes must be iteratively performed. Consequently, a large amount of calculation on correlation measurement can be prevented. Nevertheless, due to the structural simplicity, several deficiencies and shortcomings remain in the optimized linear clustering framework in SNIC:

- Redundant computations still exist in superpixels during region growing, since boundary pixels might be inspected more than once;
- The greedy strategy performed in a priority queue is prone to premature convergence;
- Once there is no global iterative updating, the single online average clustering is vulnerable to seed initialization.

Apart from those problems, the unchanged SLIC-like correlation measurement still comes to a compromise on the abovementioned contradiction. In practice, it is easy to violate color homogeneity, especially in complicated and textured regions.

Conventionally, both SLIC and SNIC superpixels have already achieved state-of-the-art (SOTA) segmentation results. In practical applications, the performance could be better promoted if the algorithms could address the abovementioned bottlenecks. To this end, a novel superpixel decomposition framework is proposed in this paper and is referred to as hybrid linear clustering (HLC). Instead of elaborating on the study of new features or measurements, HLC adopts the structured analysis and design of existing work. It inherits both the pixel clustering accuracy of SLIC and the label updating efficiency of SNIC while ameliorating the shortcomings within each single framework. The major contributions from this study can be listed as follows:

- Different from grid sampling seeds for clustering pixels, the convergence results of a SLIC-like iterative clustering structure are utilized as inceptions for superpixel region clustering in the first stage. Accordingly, new centers are context-aware without falling into local optimum trap;
- Inspired by the process of online average clustering in SNIC, an efficient region-growing based label expansion structure is proposed to generate superpixels in a one-pass manner. Substantially, it is a series of re-labelling operations on the clustering results of the previous stage;
- To further reduce the computational cost of conventional linear clustering frameworks, two acceleration strategies are proposed for iterative and online average clustering, respectively. Consequently, the efficiency of optimized structures can be both guaranteed;
- The stage-by-stage clustering processes hybridize an integrated framework, wherein the two stages generate a synergistic effect to produce higher-quality superpixels. Compared with other state-of-the-art methods, it is evenly matched in terms of segmentation accuracy, spatial compactness and running efficiency.

The rest of this paper is organized as follows. Several related SLIC-like variants are reviewed in Section 2. Section 3 explains the proposed two-stage segmentation framework in detail. The qualitative and quantitative analyses are presented in Section 4. Section 5 displays a potential application for traffic scene analysis and Section 6 gives a conclusion and perspective.

## 2. Related Works

In addition to SNIC, SLIC comes up with several enlightening principles that also have been extended in other subsequent works. In recent years, there is a large amount of literature about optimizing the linear clustering framework [12,13,14,15,16,17,18,19]. Generally, they can be divided into three categories: feature optimizations, structure optimizations and clustering optimizations.

### 2.1. Feature Optimizations

Conventional SLIC utilizes a joint color–spatial measurement to calculate the correlation between a pixel and the corresponding cluster center. Since the 3-dimensional color space and 2-dimensional position space are irrelevant in information distribution, a normalized factor is introduced to standardize them in a 5-dimensional Euclidean space. As a hyper-parameter, the factor weighs the influence of color/position on superpixel homogeneity/compactness. 

The work in [12] draws on the idea of joint measurement to guide the watershed-based region growing process, thus generating spatially compact superpixels. As a substitute for 3-dimensional CIELab color in SLIC, the conventional gradient information is extended with distance awareness in the proposed watershed superpixels (WS). In this way, watershed-based over-segmentation acquires the fundamental property of spatial compactness [13]. Nevertheless, an inadequately normalized factor may deteriorate the trade-off between segmentation precision and visual quality [14]. To this end, several SLIC variants optimize themselves from the perspective of feature space and similarity distance to achieve content-aware correlation measurements. Giraud et al. [15] adopt a multi-dimensional information fusion pattern that takes color and spatial and contour information into consideration. In the proposed superpixels with contour adherence using a linear path (SCALP) algorithm, the pixel-cluster correlation is decided by a joint effect of the 5-dimensional color–spatial feature and 1-dimensional contour intensity. The results are reported with not only regularity in sizes and shapes but maintenance of regional color homogeneity as well.

### 2.2. Structure Optimizations

As mentioned above, a typical structural optimization work is SNIC, which substitutes iteration-based updating to online dynamic updating. Furthermore, another experience is in reducing the iteration cost, that is, eliminating the redundant calculation during a series of iterations.

Choi et al. [16] introduce the inter-pixel redundancy to chop unnecessary measurements of pixel–cluster correlations in each iteration of SLIC. They believe that if a pixel’s neighboring elements are associated with a common cluster, the correlation is about the same to itself. Therefore, they perform SLIC on the subsampled image and then adopt an adaptive interpolation to fulfill the label map. As a result, the running efficiency is significantly improved. Fast linear iterative clustering (FLIC) [17] accelerates SLIC via a novel active search mechanism and a back-and-forth traversal strategy based on neighboring continuity. The proposed active search mechanism weakens the restraint of fixed search ranges in restricting local k-means. The back-and-forth traversal strategy can thereby achieve a wide range of candidate pixel inspections. Compared with SLIC, the clusters converge with fewer iteration times.

### 2.3. Clustering Optimizations

Despite the restricted local k-means clustering being straightforward, it is observed that the data should be linearly separable, and only in this situation can it work better. To remove the limitations of image information, several other clustering algorithms are introduced to generate superpixels. 

Shen et al. [18] adopt a split-and-merge manner to efficiently cluster pixels into groups, and then merge the small clusters into adjacent homogenous superpixels. Different from k-means, the proposed DBSCAN executes a non-iterative clustering operation, termed density-based spatial clustering of applications with noise. The overall algorithm achieves a satisfactory real-time performance. In the work of [19], superpixel decomposition is modeled by a pixel-related Gaussian mixture model (GMM). Specifically, each pixel is modeled by a weighted sum of Gaussian functions, and the function is associated with a superpixel. The parameters are then calculated via expectation maximization (EM). The results report that GMM superpixels perform better on complex image content than SLIC.

## 3. Proposed Framework

**Figure 1:**
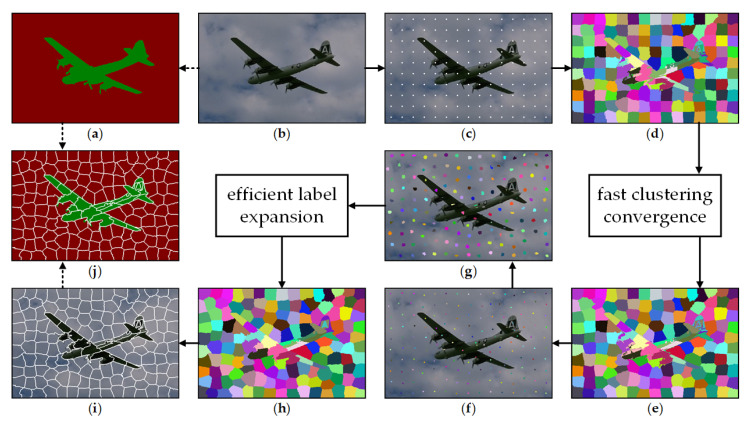
Workflow of the proposed HLC superpixel decomposition framework. (**a**) Ground truth of input image (**b**); (**b**) Input image; (**c**) Grid sampling seeds initialization; (**d**) Label map after the first iteration of SLIC, wherein different colors represent different label values; (**e**) Accelerated clustering result of SLIC; (**f**) Relocated centers of clusters of (**e**); (**g**) Region-growing-based label expansion from centers in (**f**); (**h**) Accelerated result of (**g**); (**i**) Result of HLC superpixel decomposition—the outlines in white are boundaries of different labels; (**j**) Ground truth covered by HLC superpixels.

Figure 1 provides a schematic illustration of the proposed HLC framework, wherein the key idea is to band the performance advantages of iterative and online average clustering together. To facilitate accurate pixel classification, a novel initial center location method is proposed for clusters, which is performed by a SLIC-based fast clustering convergence procedure. The relocated centers are then fixed as the starting points for generating superpixels based on an efficient region-growing-based label expansion. This two-stage framework could yield a synergistic framework that overcomes several intrinsic drawbacks in a conventional linear clustering framework, thus providing a fundamental basis for desirable segmentation results.

### 3.1. Fast Clustering Convergence

From the point of view of pixels classification, SLIC adopts a restricted k-means approach to iteratively update the clustering centers from an original state with an even distribution on the image plane. The major procedure contains the following five steps:

1. Modeling: The joint 5-dimensional feature of a pixel $I_{i}$ in an image $I={\left\{I_{i}\right\}}_{i=1}^{N}$ with N elements in CIELab color space can be modeled as $F\left(I_{i}\right)=\left[C\left(I_{i}\right),P\left(I_{i}\right)\right]$. Specifically, $C\left(I_{i}\right)=\left[l\left(I_{i}\right),a\left(I_{i}\right),b\left(I_{i}\right)\right]$ and $P\left(I_{i}\right)=\left[x\left(I_{i}\right),y\left(I_{i}\right)\right]$ represent the 3-dimensional color feature and 2-dimensional position feature of $I_{i}$, respectively.

2. Initialization: A set of seeds ${\left\{s_{k}\right\}}_{k=1}^{K}$ is evenly sampled from K grids in I, wherein K is a hyper-parameter that expects the superpixel number. That is, I is initially partitioned to K regular grids with a step of $S=\sqrt{N/K}$.

3. Inspection: For each seed $s_{k}$ , it acts as the initial cluster center $\Omega_{k}$ and then searches its 2S×2S square context region to calculate the correlation distance with the unlabeled pixels $I_{i}$ therein

(1) $$D\left(I_{i},\;s_{k}\right)=\sqrt{{\left\|C\left(I_{i}\right)-C\left(s_{k}\right)\right\|}_{2}^{2}+\lambda^{2}{\left\|P\left(I_{i}\right)-P\left(s_{k}\right)\right\|}_{2}^{2}}\text{,}$$

where λ is the normalized factor to balance color and spatial proximity, and ‖ ⋅ ‖ represents the Euclidean norm;

4. Assignment: Once $I_{i}$ is inspected by all cluster centers whose search region contains itself, it acquires the same label of $\hat{s_{k}}$ with the maxima correlation, i.e.,
  (2) $$L\left(I_{i}\right)=L\left(\hat{s_{k}}\right),\;\hat{s_{k}}=\arg\;\min\;D\left(I_{i},s_{k}\right)\text{;}$$

5. Updating: Once all pixels within I are labeled, the cluster centers $s_{k}$ are updated via k-means averaging

(3) $$F\left(C\left(s_{k}^{\left(1\right)}\right),\;P\left(s_{k}^{\left(1\right)}\right)\right)=\sum_{i\in \Omega_{k}}\left[C\left(I_{i}\right),P\left(I_{i}\right)\right]\;/\;\left|\Omega_{k}\right|,\text{,}$$

where $\left|\Omega_{k}\right|$ is the number of pixels in $\Omega_{k}$. This procedure is performed on all K clusters, which adjust ${\left\{s_{k}\right\}}_{k=1}^{K}$ to ${\left\{s_{k}^{\left(1\right)}\right\}}_{k=1}^{K}$ (the bracketed number in the corner mark of $s_{k}$ indicates the iteration time). In the next iterations, pixels would be associated with new labels based on step 3 to 4, and the new cluster centers are updated. This step is repeated until all clusters converge.

Theoretically, a good seed initialization is of benefit to any clustering framework. In a seed-demand superpixel algorithm, it is also advantageous to superpixel clusters that agglomerate from several ideal inception points [20]. Nevertheless, SLIC merely adopts grid sampling to initial its seeds (Figure 1c), which requires several updating iterations to converge all superpixel regions. It is worth noting that the overall procedure of SLIC can be re-examined as a packaged seed redistribution method since the output cluster centers could generalize the context information accurately. That is, SLIC can be regarded as a seeding step that serves another clustering structure with higher efficiency (Figure 1f). To this end, the conventional SLIC is recast for a lower computational burden while maintaining comparable performance.

As illustrated in Section 2.2, a straightforward insight for accelerating SLIC is to eliminate the redundant calculation during the iterations. In this paper, an acceleration optimization strategy is proposed through this pattern, which achieves an extreme boost on the running efficiency of SLIC. Specifically, for each cluster center $s_{i}\in {\left\{s_{i}\right\}}_{i=1}^{K}$, there is a spatial bias from the initial potion after the first iteration of SLIC

(4) $$B\left(s_{k}^{\left(1\right)},\;s_{k}^{\left(2\right)}\right)={\left\|P\left(s_{k}^{\left(1\right)}\right)-P\left(s_{k}^{\left(2\right)}\right)\right\|}_{2}\text{.}$$

What follows is a new loop, and a new $B\left(s_{i}^{\left(2\right)},\;s_{i}^{\left(1\right)}\right)$ is acquired after the second iteration. Both values are temporarily stored to guide the following updating of $\Omega_{k}$, which obeys the following two rules:

- If $B\left(s_{i}^{\left(2\right)},\;s_{i}^{\left(1\right)}\right)\geq B\left(s_{i}^{\left(1\right)},\;s_{i}\right)$ then $\Omega_{k}$ keeps active and continues the SLIC procedure in the next loop;

- If $B\left(s_{i}^{\left(2\right)},\;s_{i}^{\left(1\right)}\right)<B\left(s_{i}^{\left(1\right)},\;s_{i}\right)$ , then $\Omega_{k}$ is marked with the state of pre-convergence. Furthermore, if all adjacent clusters of $\Omega_{k}$ are concurrently pre-convergent, then $\Omega_{k}$ is marked with the state of convergence. In this case, all elements in $\Omega_{k}$ are settled with the current label and excluded from the subsequent loops.

**Figure 2:**
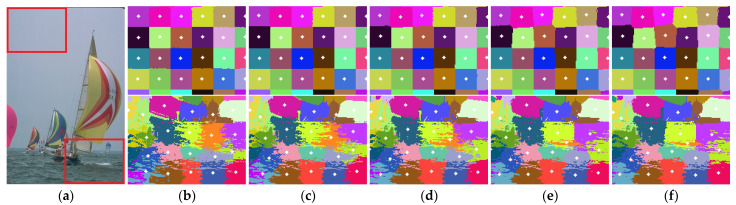
Visual comparison of clustering results by SLIC. (**a**) Input image; (**b**–**f**) Alternating columns show the zoom-in results of (**a**) after different iteration times, wherein the upper and lower column represent the label distribution within the top-left and bottom-right rectangle, respectively; (**b**) 1 iteration; (**c**) 2 iterations; (**d**) 3 iterations; (**e**) 5 iterations; (**f**) 10 iterations.

The strategy originates from the following visualized observation and analysis within the whole procedure of SLIC in Figure 2. In some detailed image regions (e.g., object boundaries and high contrast in the bottom-right of Figure 2a), the distribution of pixel labels changes distinctly within shape and size, which are very likely to be updated in the next iteration. On the other hand, several clusters barely change from being initialized to global convergence (e.g., flat background on the top-left), whereas they could still preserve content homogeneity and spatial compactness. Therefore, it is feasible for these clusters to be excluded in subsequent operations.

To quantitatively describe the degree of superpixel change, the spatial bias that measures the difference of a cluster center before and after an iteration is explicitly utilized. On this basis, if the bias becomes greater, the corresponding cluster is identified as a divergence that should be further updated (rule 1). On the contrary, if the bias decreases, the cluster is inclined to converge. A more restricted condition is that the converged state of adjacent regions is concurrently the same as the central superpixel. In this case, the central superpixel can be excluded in subsequent operations, since the labels of candidate pixels in this region are almost unchanged (rule 2). Notice that the restriction concerns extra context variance, therefore, it could effectively prevent superpixels from being wrongly excluded that are prematurely converged.

### 3.2. Efficient Label Expansion

**Figure 3:**
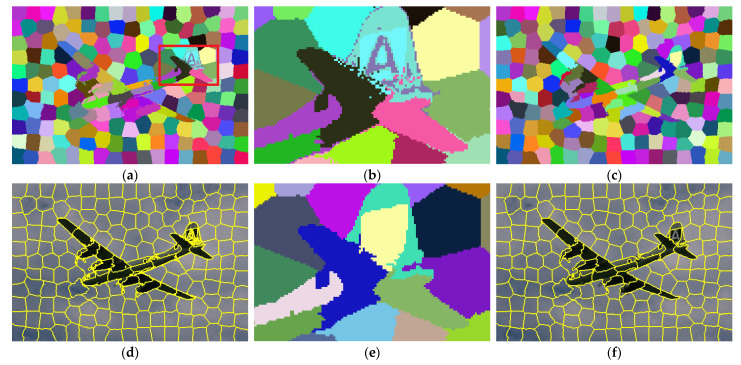
Visual difference between SLIC with and without post-processing. (**a**) Label map from SLIC without post-processing; (**b**) Zoom-in performance of (**a**); (**c**) Label map from SLIC with post-processing; (**d**) Superpixels generated by (**a**); (**e**) Zoom-in performance of (**c**), which corresponds to (**b**); (**f**) Superpixels generated by (**c**).

In practice, there is a post-processing step before cluster regions become superpixels in SLIC. Theoretically, other than geodesic distance, the proposed correlation measurement in Equation (1) cannot guarantee the spatial connectivity of pixels with identical labels [21]. For example, in Figure 3a,b, pixels on the tail of the airplane are discrete, especially on logo A. As a result, superpixels described by these clusters are spatially tangled (Figure 3d). In contrast, the merging operation could enforce spatial compactness. Nevertheless, it loses the detailed information, which results in content heterogeneity (Figure 3e,f). In fact, the process is rigid, wherein an isolated pixel is directly absorbed by its largest adjacent superpixel without correlation assessment.

This work refactors the merging post-processing in SLIC into a region-growing-based label expansion procedure that could both enforce spatial connectivity and preserve the detailed image information. The foundation of the proposed label expansion is online average clustering in SNIC, which inherits the pattern of modeling and initialization in SLIC and optimizes the inspection, assignment and updating process. The procedure can be briefed as follows.

1. Initialization (additional): A small-root priority queue Q is introduced, which returns the element with a minima key value. The grid sampled seeds ${\left\{s_{k}\right\}}_{k=1}^{K}$ are pushed in Q with zero key value;

2. Inspection: For each seed $s_{k}$ , it acts as the initial growing point $g_{k}$ of the cluster $\Omega_{k}$ and then checks the four neighboring pixels to calculate the correlation distance $D\left(I_{i},\;s_{k}\right)$ with the unlabeled one $I_{i}$ through Equation (1). The distance is then recorded as the key value of $I_{i}$ in Q with a temporary label $L\left(g_{k}\right)$

3. Assignment: The top-most element $\hat{I_{i}}$ is popped from Q, and the latest temporary label is settled, i.e., $L\left(\hat{I_{i}}\right)=L\left(\hat{g_{k}}\right)$,
  (5) $$\hat{I_{i}}=\arg\;\min\;D\left(\hat{s_{k}},I_{i}\right),\;\hat{s_{k}}\in {\left\{s_{k}\right\}}_{k=1}^{K},I_{i}\in Q\text{,}$$
  where $\hat{g_{k}}$ is the growing point of cluster $\hat{\Omega_{k}}$ centered at $\hat{s_{k}}$. Notice that ${\left\{s_{k}\right\}}_{k=1}^{K}$ are the first K popped elements before any non-seed pixels since their key value is initialized with a global minimum.

4. Updating: $\hat{I_{i}}$ becomes a steady member of the cluster $\hat{\Omega_{k}}$, and then upgrades the center $\hat{s_{k}}$ to $\hat{{s^{\prime }}_{k}}$ as follows
  (6) $$F\left(\hat{{s^{\prime }}_{k}}\right)=\left(\sum_{i\in \hat{\Omega_{k}}}\left[C\left(I_{i}\right),P\left(I_{i}\right)\right]+\left[C\left(\hat{I_{i}}\right),P\left(\hat{I_{i}}\right)\right]\right)/\left(\left|\hat{\Omega_{k}}\right|+1\right)\text{.}$$

In addition, it also acts as a new growing point of $\hat{{\Omega^{\prime }}_{k}}$ (the upgraded $\hat{\Omega_{k}}$) to execute the inspection in the next loop.

Steps 3 and 4, which are jointly executed, establish the online averaging process in SNIC. During the subsequent operations, this process is repeated until Q is empty. Compared with SLIC, the iteration-based global label re-assignment is converted to sorting-based local label expansion. Nevertheless, this conversion results in many pixels being inspected more than once, since each of them might be adjacent to different growing points before its label is settled. This often occurs within adjacent superpixels, where the boundary pixels are checked at least two times.

Regardless of that drawback, conventional SNIC adopts a greedy strategy to locally select a similar pixel for a cluster. It is required for this work to set up a priority queue so that the similarity measurement can be preserved for sorting. On the other hand, the computation and memory complexity will increase with a greater superpixel number. Meanwhile, SNIC must dynamically update cluster centers since it works in a region-growing pattern, otherwise all clusters are prone to prematurely converge. However, the variation of a cluster center usually affects the similarity measurement. For example, if there are two potential candidate pixels with identical correlation distances to a cluster center, the correlation tends to be different once the cluster updates itself by absorbing the third element; this means that the inspection step is out of sync with the joint assignment and updating, which may accumulate the misclassification of unlabeled pixels.

A special case is that the centers of all clusters are pre-defined, which avoids being upgraded by sequentially absorbing neighboring pixels via region growing. Therefore, an unlabeled pixel can be directly assigned a settled label once it is inspected by the first growing point—without enqueueing and dequeueing. This operation has a reciprocal interpretation of clustering. Given the converged state as prior knowledge, it can be regarded as a backtrack of conventional SNIC, wherein the correlation distance from an unlabeled pixel to the cluster center is a fixed value. In this way, the center-fixed online average clustering in SNIC becomes more like watershed transformation, which could efficiently work in a one-pass manner.

### 3.3. Hybrid Implementation

As discussed above, there are two strategies for optimizing the conventional linear clustering framework for different orientations. One is the fast-clustering convergence for iterative clustering, which can be utilized as an efficient tool to generalize the information on an image plane. Another is the efficient label expansion that enables the conventional online averaging to be a one-pass label assignment process. The two accelerated structures can be synergized as a concise yet integrated clustering-based superpixel generation framework, which yields considerable improvements from part to whole.

First, the key idea of fast-clustering convergence is that not all clusters need continual updating during global loops in SLIC, which results in two-fold positive effects:

- The number of clusters that participate in global updating gradually decreases within each loop, since the pre-converged superpixels are increasing. Compared with the original framework, the computational efficiency can be significantly boosted;
- The criterion of global iteration termination becomes adaptive for different input images and parameters. As a substitute, the overall iteration process could dynamically terminate itself only if all superpixels are marked as pre-converged.

In addition, the benefit of efficient label expansion is also desirable. The consecutive inspecting–assigning–updating operation effectively eliminates the inspected-yet-unlabeled elements in the priority queue, since each pixel would be inspected only once. As a result, the computation and memory complexity are drastically reduced.

Finally, the synergistic implementation works as a two-stage clustering re-converging pattern. It maintains a satisfactory property of context generalization while being executed more efficiently; moreover, the merging post-process is substituted with a one-pass label assignment process, which could lead the region-growing to achieve better homogeneity. Both strategies build the foundation to generate outstanding superpixels, which are exhibited and evaluated in the next section. 

## 4. Experiments and Discussions

This section validates and analyzes the proposed HLC framework in detail. First, the dataset, benchmark and comparison algorithm are introduced (Section 4.1). Secondly, the optimization of each strategy on HLC is discussed (Section 4.2). Moreover, the synergistic performance is compared with several state-of-the-art algorithms (Section 4.3).

### 4.1. Experiment Setup

In this part, all experiments are tested on a well-known segmentation dataset, Berkeley Segmentation Data Set 500 (BSDS500) [22]; it comprises three subsets—a training (100), validation (200) and testing (200) set—wherein each image is 481×321 or 321×481 in size, along with manual ground truth. HLC is implemented with C++ and compared with SLIC and SNIC on an Intel Core i7-7700 (4.2 GHz) and 16 GB RAM personal computer. In addition, several state-of-the-art superpixel algorithms introduced in Section 2 are utilized as references, including SCALP [15], FLIC [17] and DBSCAN [18] with different types of optimizations. Meanwhile, the conventional SLIC and SNIC are also compared as baselines. For the sake of fairness, all experiments are executed on a common benchmark [23]. The compared algorithms are all based on publicly available code with default parameters on BSDS500. Table 1 shows the detailed properties, including the controllability of superpixel number, controllability of shape compactness, the iterability of the algorithm (whether iteration is needed), computational complexity and code implementation.

To evaluate the segmentation results objectively, four evaluation metrics in [23] are considered, namely boundary recall (BR), under-segmentation error (UE), achievable segmentation accuracy (ASA) and shape compactness (SC). All of them are commonly used in superpixel decomposition methods with an emphasis on edge consistency, region homogeneity, segmentation and visual quality and the performance of subsequent visual tasks, respectively. Given the calculated superpixels $\Omega ={\left\{\Omega_{k}\right\}}_{k=1}^{K}$ and the corresponding ground truth $G={\left\{G_{m}\right\}}_{m=1}^{M}$ of an image ${\left\{I_{i}\right\}}_{i=1}^{N}$, those metrics are mathematically defined as follows.

BR is a popular metric that evaluates contour consistency, which predicts that the subsistent boundary of objects in an image should be depicted by the outlines of several gathered superpixels as consistently as possible. The value is the ratio of ground truth boundaries covered by superpixel boundaries

(7) $$\mathrm{BR}\left(\Omega ,\;G\right)=\frac{\sum_{i\in G_{b}}B\left(\min_{j\in \Omega_{b}}{\left\|P\left(I_{i}\right)-P\left(I_{j}\right)\right\|}_{2}<r\right)}{G_{b}}\text{,}$$

where $\Omega_{b}$ are the outline pixels in Ω, and $G_{b}$ are the boundary pixels in G; therefore, a greater BR value indicates a closer consistency from the superpixel edge to real image boundaries. 

UE measures how each superpixel overlaps with only one object. It is the ratio of the leaked pixels to the actual segmented pixels, wherein the former refers to the pixels beyond the intersection of the superpixel and the ground truth.

(8) $$\mathrm{UE}\left(\Omega ,\;G\right)=\frac{\sum_{m=1}^{M}\left(\sum_{\Omega_{k}|\Omega_{k}\cap G_{m}\neq \phi }\left|\Omega_{k}\right|\right)-N}{N}$$

Compared with BR, it utilizes segmentation regions instead of boundaries for measurement, which is negatively correlated with segmentation accuracy.

ASA describes the accuracy of segmentation results. It reveals the percentage of the correct segmentation in terms of the ground truth,

(9) $$\mathrm{ASA}\left(\Omega ,\;G\right)=\frac{\sum_{k=1}^{K}\arg\max_{m}\left|\Omega_{k}\cap G_{m}\right|}{\sum_{m=1}^{M}\left|G_{m}\right|}\text{,}$$

and also uses region information to evaluate the performance, as with UE. A higher ASA value indicates that the performance of superpixels in subsequent iterations are unaffected [24].

SC describes the roundness of each superpixel patch, as depicted in Equation (10), and it is positively associated with the regularity and uniformity of the superpixel shape.

(10) $$\mathrm{SC}\left(\Omega ,\;G\right)=\frac{1}{N}\sum_{k=1}^{K}\left|\Omega_{k}\right|\frac{4\pi A\left(\Omega_{k}\right)}{P\left(\Omega_{k}\right)}.\text{.}$$

Generally, a higher overall SC corresponds to a better topology-preserved distribution of superpixels on the image plane.

### 4.2. Synergistic Effect Analysis

As mentioned above, HLC is essentially composed of two optimized structures based on SLIC and SNIC, which works stage-by-stage to generate a synergistic effect. This subsection conducts a series of experiments to compare the improvements in conventional SLIC as well as SNIC. For concise representation, the proposed fast clustering convergence and efficient label expansion are termed FCC and ELE, respectively, later in this paper.

**Figure 4:**
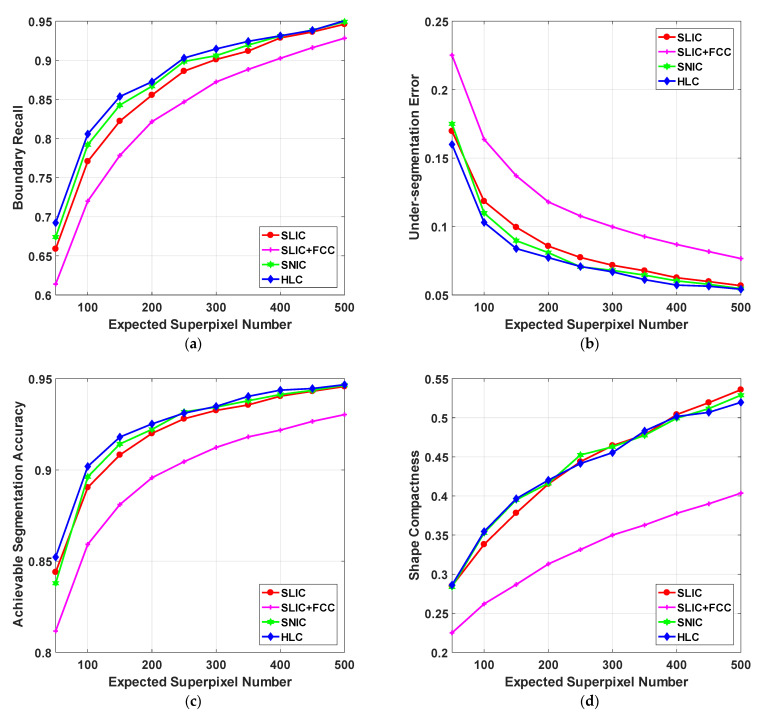
Quantitative evaluation of improvements on conventional SLIC and SNIC on the testing subset of BSDS500. (**a**) Boundary recall; (**b**) Under-segmentation error; (**c**) Achievable segmentation accuracy; (**d**) Shape compactness. The expected superpixel number ranges from 50 to 500.

**Figure 5:**
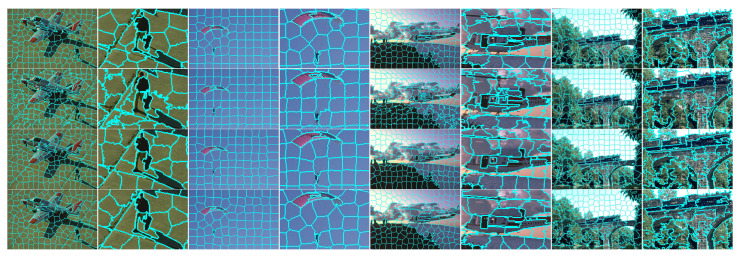
Visual comparison of improvements on conventional SLIC and SNIC. Superpixels from top row to bottom are generated by SLIC, FCC-optimized SLIC (SLIC + FCC), SNIC and HLC. The excepted number of superpixels is fixed to 200. Alternating columns show each segmented image followed by local details.

The inferior curve of SLIC + FCC in Figure 4 indicates that it suffers a downside by merely introducing FCC to the conventional SLIC framework. Accordingly, the intuitive insight of quantitative results is significantly inferior, despite the baselines not being desirable enough. For example, there are more misclassifications occurring in object boundaries compared with SLIC in Figure 5 (row 1 and 2), especially for twigs and small objects.

On the other hand, the integrated HLC profits from SNIC-based ELE make up for the performance loss by FCC. Intrinsically, the misclassified pixels are re-measured with an expanding cluster, which maintains higher homogeneity than that in SLIC. Therefore, superpixel regions that are wrongly pre-converged by FCC can be further fine-tuned. Furthermore, incorrect boundaries caused by the rigid merging post-process can also be radically avoided. Compared with SNIC, an HLC superpixel originates from the position that holds an average eigenvalue of spatial context, which is superior to a grid-sampled seed with discrete image information. In this way, adjacent cluster regions are more sensitive to low contrast and complex textures. As shown in Figure 4, the results of HLC are equivalent, and sometimes even better than the other two baselines. To sum up, it is accepted that the two strategies could generate a synergetic effect that desirably optimizes the segmentation results, wherein several inherent drawbacks in the conventional linear clustering framework are ameliorated. 

Table 2 lists the execution time of HLC compared with SLIC and SNIC stage-by-stage, along with the corresponding optimizations, FCC and ELE, respectively. The execution time of each algorithm is averaged by 200 testing images with 200 expected superpixel numbers (more details are discussed in the next subsection). 

Specifically, HLC keeps the grid sampling of SLIC (11 ms) and then utilizes the clustering results of FCC (16 ms) as the inception, followed by performing ELE (11 ms) to eventually generate superpixels (37 ms). Specifically, the clustering efficiency within SLIC is more than doubled by FCC. In addition, ELE could free up approximately two-thirds of the clustering time in SNIC, which can be regarded as an optimized post-processing step for FCC-based SLIC. As a result, this stage-by-stage implementation is cost-efficient, and the overall execution time of HLC superpixel generation is less than either SLIC or SNIC.

### 4.3. SOTA Comparisons

**Figure 6:**
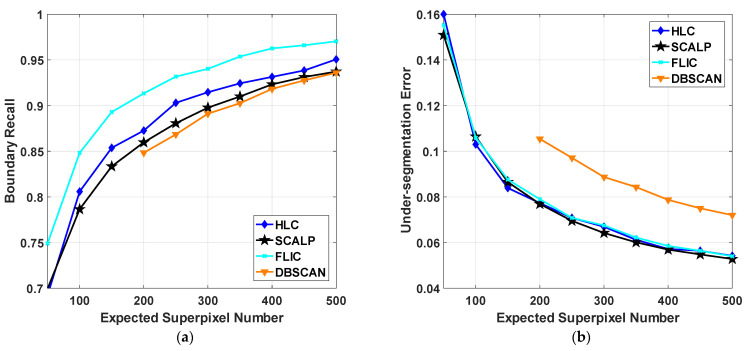

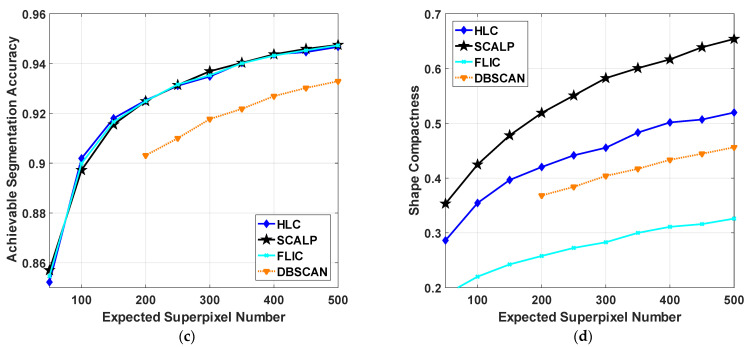
Quantitative evaluation of four algorithms on the testing subset of BSDS500. (**a**) Boundary recall; (**b**) Under-segmentation error; (**c**) Achievable segmentation accuracy; (**d**) Shape compactness. The expected number of superpixels ranges from 50 to 500. The curve of DBSCAN is incomplete since its available implementation is not robust to some hyper-parameters.

**Figure 7:**
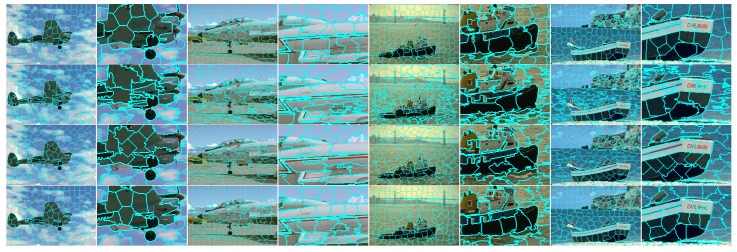
Visual comparison of segmentation results with 200 expected superpixels. Superpixels from top to bottom are generated by SCALP, FLIC, DBSCAN and HLC. Alternating columns show each segmented image followed by local details of each image.

The primary goal of HLC is to integrate the existing clustering-based work rather than typical principal improvements. Therefore, feature-oriented SCALP, structure-oriented FLIC and clustering-oriented DBSCAN with single optimization are compared to verify the superiority of the hybrid clustering structure. Quantitative and qualitative results are visualized in Figure 6 and Figure 7, respectively.

Figure 6 shows the quantitative evaluation of all methods. Specifically, FLIC acquires the best BR at the expense of the worst SC. Despite the fact that the active search strategy could avoid clusters being limited to a fixed range in space and thus achieving better boundary adherence and region homogeneity, the constraint of spatial correlation is neglected by FLIC. In practice, the outlines of FLIC superpixels are irregular and sinuous, resulting in the generated superpixels being too chaotic to analyze (row 2 of Figure 7). Apart from FLIC, many other superpixel algorithms with superfluous boundaries are reported to easily lose controllability on shape regularity [25]. Furthermore, since the false boundary detection is unconcerned with BR, the computational burden on superpixel merging in these algorithms are usually much heavier [26].

HLC achieves comparable performance with SCALP and FLIC in terms of UE and ASA. By fully exploiting the structural constraint within linear clustering, HLC strengthens the representation of correlation measured by color–spatial distance. Meanwhile, other than FLIC, it provides a balanced trade-off between boundary adherence and shape compactness. Compared with SCALP, which is known as a well-rounded method, HLC could also provide a satisfactory visual quality from whole to part.

In addition, benefiting from the relocation of cluster centers, HLC could effectively promote clustering accuracy more than that in other clustering frameworks. First, it overcomes a major downside of grid sampling initialization: that all inceptive seeds are poor at representing local context information; whereas in FLIC and SCALP, the ability of representation is iteratively enhanced. Compared with the seed selection method in DBSCAN, HLC could generate superpixels with more content awareness and avoid them being located on the object boundaries. As a synergetic result, HLC superpixels ensure both color homogeneity and boundary adherence while maintaining a stably regular shape.

A comprehensive discussion on the performance of execution time is presented in Table 3. In principle, SLIC, FLIC and SCALP work in a similar pattern. As a baseline framework, SLIC shows O(N) complexity, but the kernel algorithm requires iter times of iteration. Therefore, the practical complexity is O(iter∗N) [20]. FLIC could achieve fast convergence via an active search strategy, compared with SLIC, and the overall updating is merely iterated 2–3 times. Accordingly, it could run over 50% faster than SLIC with 10 fixed iterations. SCALP pursues a better comprehensive performance at the expense of time efficiency, which requires more execution time for correlation measurements of all pixels along the linear path from a cluster center. On average, it runs over 10-times slower than SLIC.

On the other hand, SNIC and DBSCAN could both generate superpixels without global iteration. Given the time complexity of priority queue sorting, the complexity of SNIC is actually O(log(n)∗N), where n is the queue length. DBSCAN adopts a split-and-merge framework that both the clustering and the merging procedure have low computational complexity, which shows O(N) complexity and achieves real-time performance on BSDS500. Nevertheless, the segmentation results are severely limited by the framework, whose performance is the worst in the comparisons in Figure 6—except for shape compactness.

Benefiting from the speed-up gains in the two-stage implementation, HLC also theoretically has O(N) time complexity. Prior to introducing the contour in a complicated path-based measurement, HLC merely adopts conventional color and spatial information to calculate a point-to-point feature distance. The efficiency advantage is outstanding—on average, HLC runs over 15-times faster than SCALP. It is also worth noting that, as the expected superpixel number increases, execution times for all the algorithms also slightly increase. Among them is HLC, which not only reduces the computation cost but lowers the additional time for an increasing superpixel number (tending to be faster than FLIC). Therefore, it can be utilized as a desirable pre-processing tool for the task at hand, which could provide a satisfactory balanced trade-off between segmentation quality and running efficiency.

**Table 3:** Comparison of execution times among different algorithms (ms).

Methods	Expected Superpixel Number									
	50	100	150	200	250	300	350	400	450	500
SCALP	571	607	614	605	605	577	573	593	565	543
FLIC	33	35	36	37	38	39	40	42	42	43
DBSCAN	-	-	-	35	34	34	33	33	33	33
SLIC	50	51	51	52	52	52	53	54	54	55
SNIC	41	41	42	43	43	44	44	45	45	45
HLC	35	37	38	38	39	40	39	40	40	40

## 5. Application

In practical applications, superpixels are utilized as region-level descriptors to improve the performance of advanced tasks. A representative work is traffic scene analysis in the field of automated vehicles [27]. To this end, the results of HLC are merged by mean shift (MS) [28] to accomplish semantic segmentation. All input images are selected from the CamVid Database [29], which consists of various real scenarios by driving recorders.

**Figure 8:**
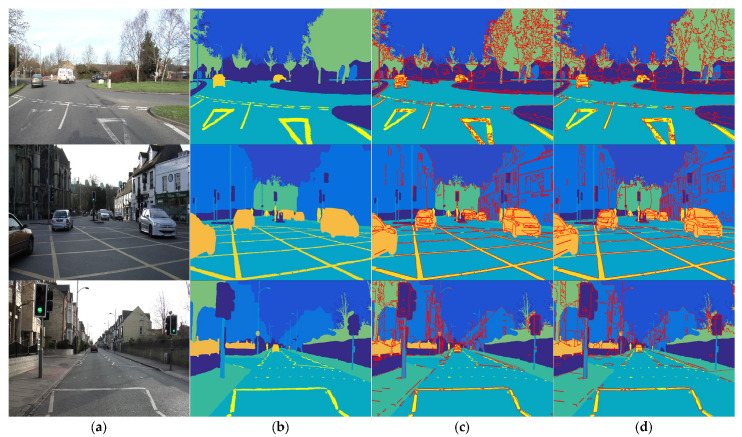
Segmentation results by applying MS to different superpixels. (**a**) Traffic scene image; (**b**) Ground-truth of (**a**); (**c**) Pixel-level MS; (**d**) HLC + MS. The excepted number of HLC superpixels is pre-set to 1000 (a larger number of superpixels may further improve the classification accuracy).

The corresponding results are illustrated in Figure 8. Theoretically, MS considers both color and spatial features in each pixel (superpixel) that achieves image segmentation by merging homogeneous neighboring pixels (superpixels) [30,31]. In practice, superpixel-level region descriptors could promote the results by eliminating image noise. As shown in Figure 8d, HLC + MS provides a much more accurate segmentation result with visual satisfaction, especially on textured regions and small targets. Essentially, the combination preserves the homogeneity of inter-superpixels and emphasizes the difference, which is based on the outstanding performance of context generalization by HLC superpixels. More importantly, it significantly reduces the computational entities within the MS framework, accelerating it by up to over 10 times for a 960×720 image. Consequently, it would be an efficient tool in the field of automated vehicles.

## 6. Conclusions

In this paper, a novel superpixel decomposition framework—termed hybrid linear clustering (HLC)—is proposed, which synergizes the properties of iterative clustering and online averaging to achieve better performance. It consists of two major optimized strategies that accelerate the conventional SLIC and SNIC for different orientations. First, the fast-clustering convergence enables the linear clustering to be more efficient, which substantially introduces two pre-convergence rules to prevent redundant computations on correlation measurements. In this way, SLIC could be performed more efficiently to generalize the information on the image plane. Other than the conventional split-and-merge process, next, an ultra-fast implementation of label expansion based on online averaging in SNIC is proposed, which is utilized as a substitute to perform clustering. The experimental results verify that the integrated framework could avoid the intrinsic drawbacks of conventional approaches and produce desirable superpixels in a limited time.

In future work, it is worth integrating more state-of-the-art algorithms to achieve better performance that emphasizes their strengths whilst circumventing weaknesses, which could make them more suitable for practical computer vision tasks.

## Figures and Tables

**Table 1:** Properties of superpixel algorithms. ✓/✗ indicates it works with/without the property.

	SCALP	FLIC	DBSCAN	SLIC	SNIC
Number	✓	✓	✗	✓	✓
Compactness	✓	✗	✓	✗	✓
Iteration	✓	✓	✗	✓	✗
Complexity	O(N)	O(N)	O(N)	O(N)	O(N)
Implementation	C++/Matlab	C/C++	C++/Matlab	C/C++	C++/Matlab

**Table 2:** Comparison of execution times at each stage within different algorithms (ms).

	SLIC	FCC	SNIC	ELE	HLC
Initialization	10	-	8	-	11
Clustering	40	16	35	11	27
Post-processing	2	-	-	-	-
Total time	52	-	43	-	38

## Data Availability

The dataset used in this work is available at: https://www2.eecs.berkeley.edu/Research/Projects/CS/vision/grouping/resources.html (accessed on 9 December 2022); The benchmark is available at: https://github.com/davidstutz/superpixel-benchmark (accessed on 9 December 2022); The source code of SLIC is available at: https://ivrlwww.epfl.ch/supplementary_material/RK_SLICSuperpixels/index.html (accessed on 9 December 2022); The source code of SNIC is available at: https://www.epfl.ch/labs/ivrl/research/snic-superpixels/ (accessed on 9 December 2022); The source code of SCALP is available at: https://github.com/rgiraud/scalp (accessed on 9 December 2022); The source code of DBSCAN is available at: http://github.com/shenjianbing/realtimesuperpixel (accessed on 9 December 2022); The source code of FLIC is available at: https://github.com/JXingZhao/FLIC/ (accessed on 9 December 2022).

## Abbreviations

SLIC	simple linear iterative clustering
SNIC	simple non-iterative clustering
SOTA	state-of-the-art
HLC	hybrid linear clustering
WS	watershed superpixels
SCALP	superpixels with contour adherence using linear path
FLIC	fast linear iterative clustering
DBSCAN	density-based spatial clustering of applications with noise
GMM	Gaussian mixture model
EM	expectation maximization
BR	boundary recall
UE	under-segmentation error
ASA	achievable segmentation accuracy
SC	shape compactness
FCC	fast clustering convergence
ELE	efficient label expansion
MS	mean shift
